# Treatments and whole exon sequencing of a case with multiple primary lung cancer

**DOI:** 10.1186/s13019-023-02161-0

**Published:** 2023-02-02

**Authors:** Guangyu Bai, Yuan Li, Ying Ji, Yue Peng, Zhenlin Yang, Liang Zhao

**Affiliations:** 1 Department of Thoracic Surgery, National Cancer Center/National Clinical Research Center for Cancer/Cancer Hospital, Chinese Academy of Medical Sciences and Peking Union Medical College, Beijing, 100021 China; 2 Department of Thoracic Surgery, Beijing Chao-Yang Hospital, Capital Medical University, Beijing, 100020 China

**Keywords:** Synchronous multiple primary lung cancers, Whole exon sequencing, Thoracic surgery, Gene detection

## Abstract

**Introduction:**

The number of patients with synchronous multiple primary lung cancer (sMPLC) has increased recently. However, diagnosing and selecting the appropriate therapeutic strategy for this type of disease is not simple.

**Case presentation:**

This report presented a case of sMPLC with lymph node metastasis. With no smoking and cancer history, this patient had seven nodules in the right lung and underwent single-portal video-assisted thoracoscopic surgery (VATS). In addition, she received four cycles of chemotherapy after the operation. Whole exon sequencing (WES) was performed in five resected tissue samples (four tumors and one lymph node). We conducted genomic profiling and clone evolution analysis of the five samples. Gene detection helped to confirm that the metastasis lymph node was transferred from one nodule. There was apparent heterogeneity of gene mutations among the five samples of the patient, with only one shared “neurofilament heavy polypeptide” (NEFH) mutation. A dominant substitution of C > T/G > A was found in all the samples. Pyclone model was used to calculate all tissues' cellular prevalence (CP) values, and NEFH mutations were thought to be the ancestral clones. During the follow-up period, residual lesions showed no apparent changes and limited response to chemotherapy.

**Conclusions:**

This report showed an essential role in genomic detection and selecting the appropriate treatment of sMPLC. Surgery remains the primary treatment strategy for this type of disease, and the occurrence and development of sMPLC need more in-depth research.

**Supplementary Information:**

The online version contains supplementary material available at 10.1186/s13019-023-02161-0.

## Introduction

With the improvement of thin section computer tomography (CT), the detection rate of synchronic multiple primary lung cancer (sMPLC) is increasing. According to the eighth edition of the American Joint Committee on Cancer (AJCC) tumor, node, and metastasis (TNM) classification, multiple primary lung nodules were classified into four types: *Type I*-Second primary lung cancers, *Type II*-Separate tumor nodules (intrapulmonary metastasis), *Type III*-Multifocal lung adenocarcinoma with ground glass/ lepidic features, and *Type IV*-Pneumonic-type lung adenocarcinoma [1, 2]. However, the diagnosis of sMPLC still needs to be careful. In recent years, molecular characteristics of multiple lesions have become widely studied for understanding such lesions’ natural lineages [3, 4]. Therefore, we always need both pathological and genomic evidence to help diagnose sMPLC [5]. For the treatment of patients with sMPLC, surgical resection was thought to be the first choice of treatment [6–8]. However, considering some nodules scattered in bilateral lung lobes, it is challenging to obtain radical resection at one time. Previous studies have demonstrated that although some patients had residue ground glass opacities (GGOs) after surgery, the overall survival (OS) remained satisfying, with a 5-year-OS rate of over 80% [9]. For non-surgical treatment, immunotherapy has been used to treat multiple GGOs. However, Zhang et al. [10] showed that immunotherapy is ineffective in treating multifocal lung GGOs. Therefore, the choice of comprehensive treatment mode for sMPLC has become a critical problem in clinical diagnosis and treatment.

In this study, we presented a case with seven nodules located in the right lung. Right lower lobe lobectomy and systematic mediastinal lymph node dissection were performed on this patient. We delivered resection samples (four tumor samples with their matched paracancerous tissue samples and one lymph node) to whole exon sequencing (WES), and presented the treatment process, prognosis, and genomic analysis results. PyClone [11] was used to cluster subclones inferred from single nucleotide variants (SNVs). The cancer cell fraction (CCF) of somatic SNVs in paired samples was calculated using PyClone, which indicates the proportion of cells in the tumor sample that harbor that mutation.

## Case presentation

**Fig. 1:**
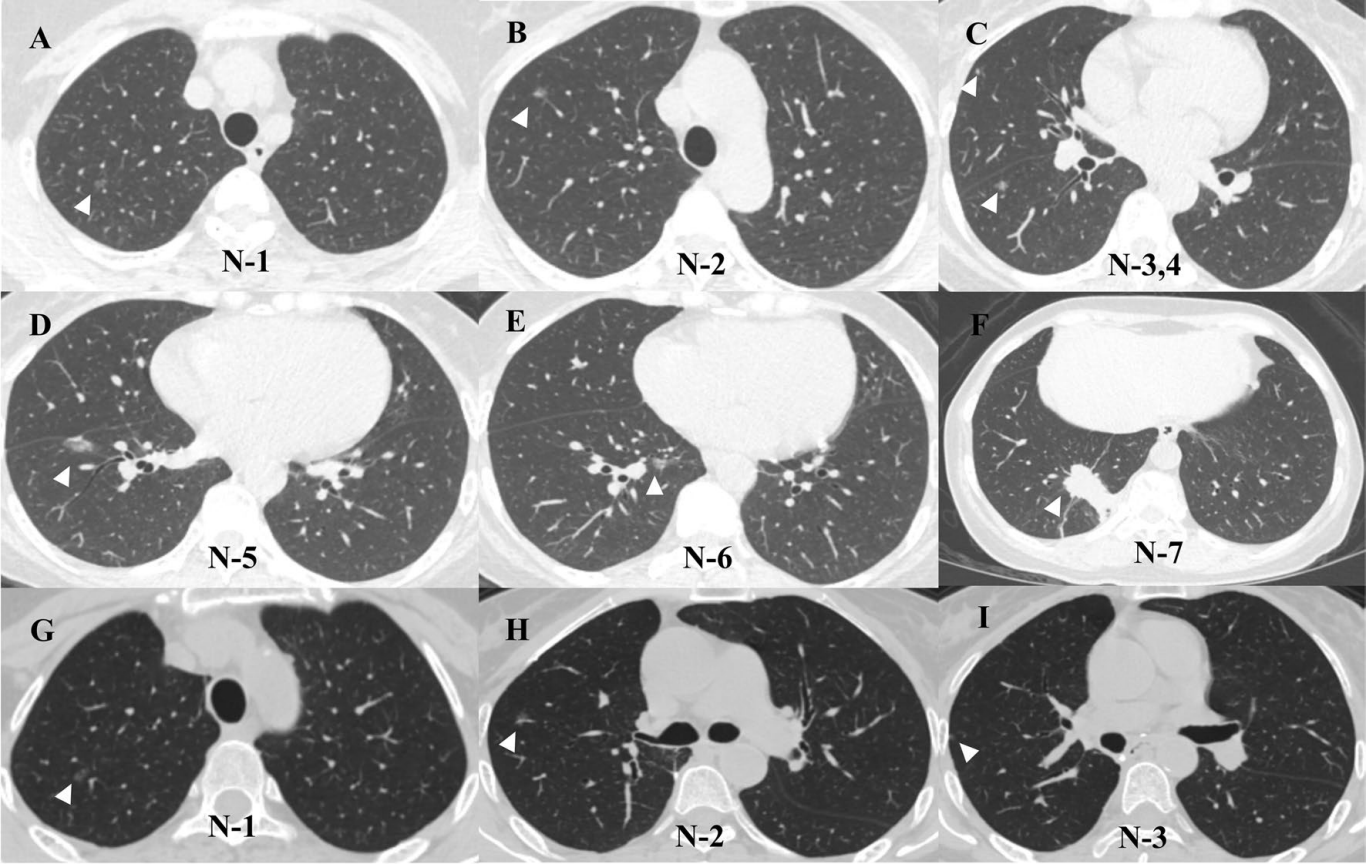
Chest CT scan finding of the patient. **A**–**F** the right upper lobe shows two 0.6 cm GGOs in S1 (**A**, **B**). the right middle lobe shows a 0.3 cm GGO in S5 (**C**). the right lower lobe shows an 0.8 cm GGO in S6 (**C**), a 1.6 cm GGO in S7 (**D**), a 1.3 cm partial solid nodule in S8 (**E**), and a 2.6 cm solid nodule in S10 (**F**). **G**–**I** Chest CT scan shows the remaining lesions' after thirty-six months of follow-up

**Fig. 2:**
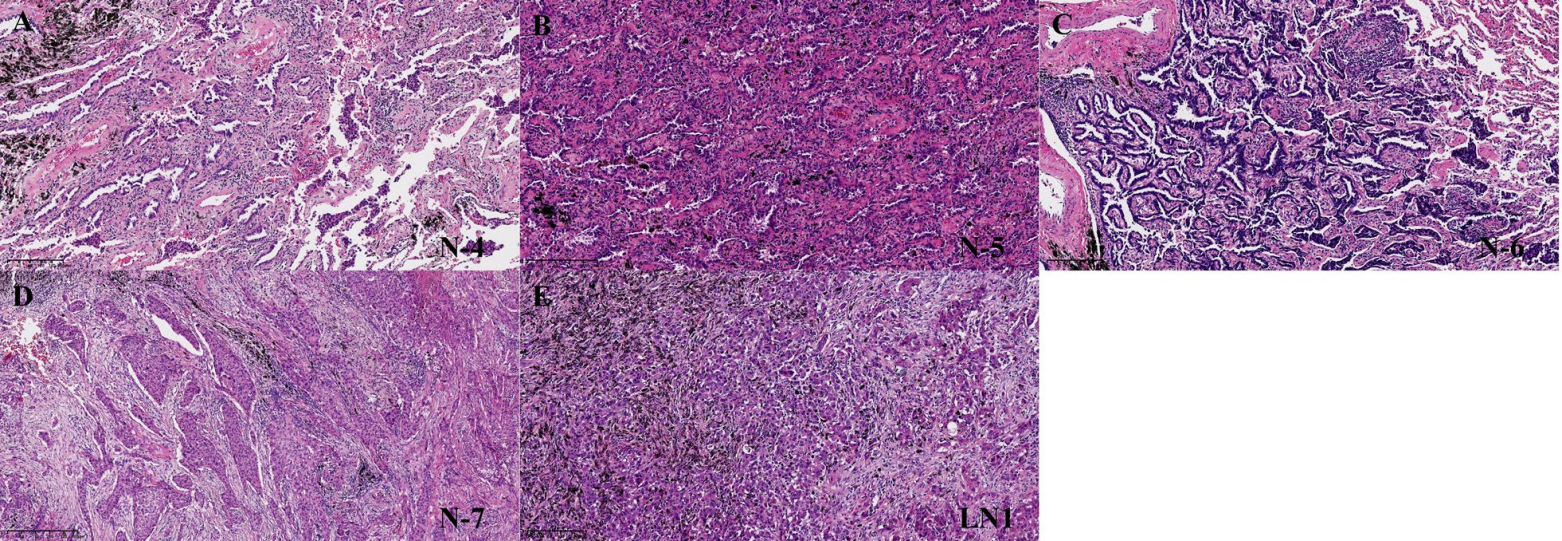
Pathological results of the resected samples (**A**–**E**). N-4 is acinar predominant lung adenocarcinoma (**A**). N-5 is papillary predominant lung adenocarcinoma (**B**). N-6 is lepidic predominant lung adenocarcinoma (**C**). N-7 is solid predominant lung adenocarcinoma (**D**). LN1 is a metastatic lymph node (**E**). All the slices were presented in hematoxylin–eosin (HE) stained 10 × magnification

A 58-year-old woman who never smoked was referred to our hospital for further diagnosis and treatment due to multiple lung nodules found in a routine examination in 2019 (Fig. 1). Pre-operative CT examination suggested seven nodules distributed in the right lung (two in the right upper lobe, one in the right middle lobe, and four in the right lower lobe, Fig. 1A–F, N-1 to N-7) and no nodule in the left lung. Figure 1 showed us that N-7 in the right lower lobe is the largest nodule with a large solid component in the lung window, which has pleural traction. Considering the tumor's maximal removal and preservation of normal lung parenchyma, we performed right lower lobe resection and systematic mediastinal lymph node dissection on this patient. After the operation, we delivered resection specimens to pathological and gene detection. Table 1 shows the CT and pathological features of the seven nodules, and Fig. 2 shows the pathological sections of the five samples. Postoperative pathology confirmed the solid nodule (N-7 in S10) as a poorly differentiated adenocarcinoma (solid predominant subtype) with a diameter of 2.5 cm (Fig. 2D). N-4 (Fig. 2A) and N-5 (Fig. 2B) were moderately differentiated adenocarcinoma with a diameter of 0.8 cm and 0.6 cm, separately. N-6 (Fig. 2C) was well-differentiated adenocarcinoma with a diameter of 1.2 cm. Metastasis was found in N1 nodes (Station 12), and it was confirmed that the positive lymph node was metastasized from N-7 through the whole exon sequencing. Four cycles of chemotherapy (Carboplatin 500 mg + pemetrexed 800 mg) were administered after surgery in October 2019. No relapse was observed and the residual nodules showed no apparent changes on the chest CT scan nearly 36 months after the chemotherapy.

**Table 1:** Imaging and pathological characters of this patients’ nodules

Name	Location	Size* (cm)	Pathological size (cm)	Pathological subtype	Pathological grade
N-1	RUL S1	0.6	–	–	–
N-2	RUL S1	0.6	–	–	–
N-3	RML S5	0.3	–	–	–
N-4	RLL S6	0.8	0.8	Acina predominant	2
N-5	RLL S7	1.6	0.6	Papillary predominant	2
N-6	RLL S8	1.3	1.2	Lepidic predominant	1
N-7	RLL S10	2.6	2.5	Solid predominant	3

*RUL* right upper lobe; *RML* right middle lobe; *RLL* right lower lobe

^*^The diameter was detected in the lung window

### Mutational spectrum and clone evolution analysis

**Fig. 3:**
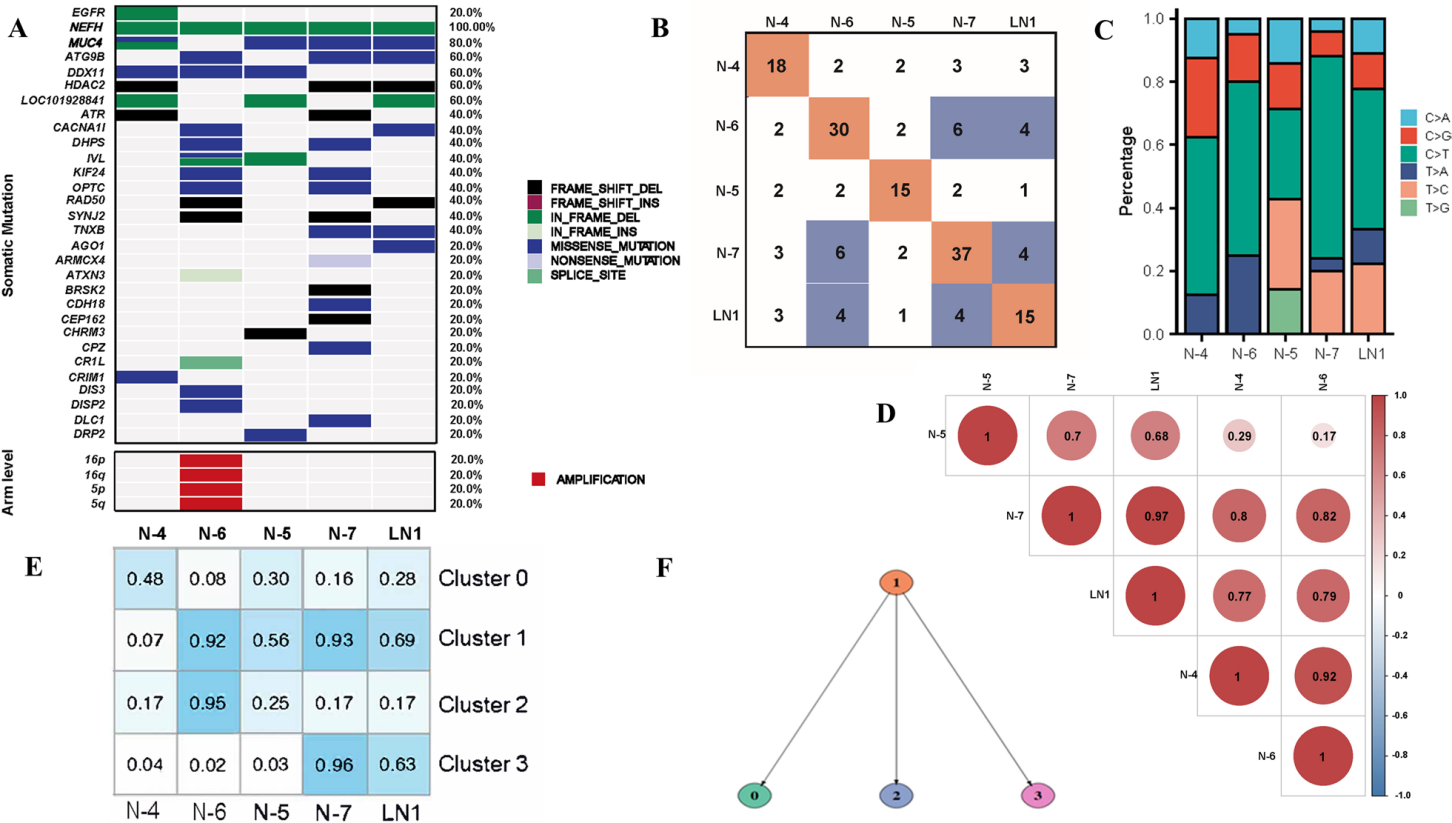
Mutational spectrum and clone evolution analysis. **A** Mutational spectrum of the five samples (only the top 30 mutated genes were shown); **B** the number of total mutations identified in each tumor (N-4 to N-7 and LN1) and the number of mutations shared by any pair of lesions; **C** the ratio of different types of mutant bases; **D** Pearson correlation coefficients of the mutation signature spectrum among the five samples; **E** CP values of the 4 clusters analyzed by PyClone; (F) Evolutional relationships of the 4 clusters

Whole exon sequencing (WES) was conducted to further analyze the relationship between the four primary tumors (N-4 to 7) and the metastatic lymph node (LN1) (Fig. 3). Among the top 30 mutation genes (results of detected actual nucleotide position/change and variant allele frequencies of the variants were provided as Additional file 1), only NEFH mutation was found in all five samples. Other mutations, including MUC4 (80%), ATG9B (60%), DDX11(60%) and HDAC2 (60%), were detected in three or more than three samples. EGFR 19del mutation was detected only in N-4. No arm-level copy number variants (CNVs) were detected except for N-6, in which 16p/q, and 5p/q harbored amplification. However, no classical lung cancer driver genes existed simultaneously in two or more samples (Fig. 3A). All five samples showed a dominant substitution of C > T/G > A, and cytosine deamination was considered in our analysis (Fig. 3C). Previous studies [12] have shown that C > A/G > T transversions were smokers' most common base conversion type, while C > T/G > A were prone to be found in non-smokers. This patient had no history of smoking, which is consistent with previous reports. Besides, 26.7% (4/15) somatic mutations of N-7 were found in metastatic lymph nodes (Fig. 3B), and metastatic lymph nodes had the most similar mutation signature (R version 3.5.3, code: https://github.com/ShixiangWang/sigminer; method: https://cancer.sanger.ac.uk/signatures/signatures_v2/) with N-7 (Pearson = 0.97) (Fig. 3D), which suggested that the metastatic lymph nodes were metastasized from N-7.

We further analyzed cancer cell fractions with PyClone [11] to understand the clonal population structure among the five samples. Somatic mutations were classified into 4 clusters (Fig. 3E). Cluster 1 (14 mutations) has the higher CP (cellular prevalence) values in N-5, 6, 7, and lymph nodes, representing the main clone. Clusters 2 and 3 are highly distributed in N-6 and N-7, respectively, indicating that these clusters’ mutations are sub-clonal mutations, which occurred later during the tumorigenesis (Fig. 3E, F). According to the results of PyClone analysis, LN1 was evolutionarily closer to N-7, which was consistent with our pathological and genomic findings.

## Discussion and conclusions

In this paper, we presented a patient with sMPLC who received operation and postoperative chemotherapy. Pathological and genomic detection helped us to diagnose the metastatic lymph node and referred us to take postoperative chemotherapy. During the follow-up period, residual lesions were observed with no changes.

From the perspective of surgical procedure, right lower lobe resection plus systematic mediastinal lymph node dissection might be a reasonable choice. Because N-7 is the largest nodule among this patient's seven nodules with pleural traction, its solid component is more than 2 cm in the mediastinal window. Pneumonectomy should be avoided because of the indolent characteristics of GGOs and preserving lung function. This clinical decision was consistent with previous studies [13, 14], which suggested that surgical resection remains the mainstream treatment of sMPLC and pneumonectomy is a risk factor associated with poor prognosis. In addition, after chemotherapy, thirty-six months of follow-up showed that these GGO lesions appeared to have no response to chemotherapy, which is consistent with previous studies and explained by the biological indolence [15].

Besides, from the results of genomic detection, we found few shared mutations between the resected samples except NEFH. This result may be due to the small sample size of genomic detection. If we detected more patients and samples, there might be inspiring results waiting for us. Previous studies informed that NEFH (neurofilament heavy) is a cancer driver which had a functional impact on KRAS and MTORC1 signialing [16]. Furthermore, Fang et al. [17] suggested that the higher expression of NEFH mutations is associated with poor prognosis in lung adenocarcinoma, and NEFH mutations were enriched in samples after TKI treatment and were associated with reduced neutrophil infiltration. However, there were few reports about NEFH mutations in sMPLC. Therefore, our results might provide a beginning to study the relationship between NEFH and sMPLC. Moreover, the clonal population structure analysis informed that these four nodules might arise from different periods and go through different tumorigenesis processes.

If we review the diagnosis and treatment of this patient, we will find that there are other possible results for this case. Diagnosing might be the most crucial point of this case. Other nodules' characteristics might remain unclear if we made preoperative puncture to N-7 and used chemotherapy instead of surgery. And intrapulmonary metastasis might not be excluded in the end. In that case, subsequent surgical treatment may still be unavoidable. This patient will face huge expenses and tremendous mental pressure. This case told us that the utility of genomic detection is vital in diagnosing sMPLC and should be encouraged for patients with sMPLC if there is any confusion in the diagnosis.

There are also limitations in our study. First, this case study may not represent the present treatment and diagnosis of patients with sMPLC. Therefore, a larger cohort study should be launched in the future. Second, due to the patient’s personal reasons, we had no immunochemical results of these nodules, which we also wanted to present in our manuscript.

In summary, for patients with sMPLC, diagnosis and treatment need to be carefully considered. Pathological detection combined with genomic detection is highly critical. For treatment, it needs to be considered individually, and the pulmonary parenchyma and lung function should be preserved as far as possible under the premise of ensuring the surgical effect.

## Supplementary Information

**Additional file 1. Supplementary Table.** Actual nucleotide position/change and variant allele frequencies of the variants.

## Data Availability

The clinical data in this article are partially available after publication, but the consent of the author is required. The sequencing data in this paper is not public.

## Abbreviations

sMPLC: Synchronous multiple primary lung cancer
VATS: Video-assisted thoracoscopic surgery
WES: Whole exon sequencing
NEFH: Neurofilament heavy polypeptide
CP: Cellular prevalence
CT: Computer tomography
AJCC: American Joint Committee on Cancer
GGOs: Ground glass opacities
OS: Overall survival
CCF: Cancer cell fraction
SNVs: Single nucleotide variants
CNVs: Copy number variants

## References

[1] Goldstraw P, Chansky K, Crowley J, Rami-Porta R, Asamura H, Eberhardt WE, Nicholson AG, Groome P, Mitchell A, Bolejack V, Staging International Association for the Study of Lung Cancer, Advisory Boards Prognostic Factors Committee, Institutions Participating, Staging International Association for the Study of Lung Cancer, Boards Prognostic Factors Committee Advisory, and Institutions Participating. The Iaslc Lung Cancer Staging Project: Proposals for Revision of the Tnm Stage Groupings in the Forthcoming (Eighth) Edition of the Tnm Classification for Lung Cancer. J Thorac Oncol 2016;11(1):39–51. 10.1016/j.jtho.2015.09.009 26762738

[2] Detterbeck FC, Nicholson AG, Franklin WA, Marom EM, Travis WD, Girard N, Arenberg DA, Bolejack V, Donington JS, Mazzone PJ, Tanoue LT, Rusch VW, Crowley J, Asamura H, Rami-Porta R. The Iaslc Lung Cancer Staging Project: Summary of Proposals for Revisions of the Classification of Lung Cancers with Multiple Pulmonary Sites of Involvement in the Forthcoming Eighth Edition of the Tnm Classification. J Thorac Oncol 2016;11(5):639–50. 10.1016/j.jtho.2016.01.024 26940528

[3] Su K, Gao S, Ying J, Zou S, He J (2018). Sequencing a super multiple synchronous lung cancer reveals a novel variant in driver gene Arid1b. J Thorac Cardiovasc Surg.

[4] Suh YJ, Lee HJ, Sung P, Yoen H, Kim S, Han S, Park S, Hong JH, Kim H, Lim J, Kim H. A novel algorithm to differentiate between multiple primary lung cancers and intrapulmonary metastasis in multiple lung cancers with multiple pulmonary sites of involvement. J Thorac Oncol 2020;15(2):203–15. 10.1016/j.jtho.2019.09.221 31634666

[5] Li R, Li X, Xue R, Yang F, Wang S, Li Y, Shen D, Sun K, Chen K, Weng W, Bai F, Wang J (2018). Early metastasis detected in patients with multifocal pulmonary ground-glass opacities (Ggos). Thorax.

[6] Hamaji M, Ali SO, Burt BM. A meta-analysis of resected metachronous second non-small cell lung cancer. Ann Thorac Surg 2015;99(4): 470–78. 10.1016/j.athoracsur.2014.11.033 25725930

[7] Waller DA. Surgical management of lung cancer with multiple lesions: implication of the new recommendations of the 8 edition of the Tnm classification for lung cancer. J Thorac Dis 2018;10(Suppl 22):S2686–S291. 10.21037/jtd.2018.04.159 PMC6178291 30345106

[8] Yang H, Sun Y, Yao F, Keke Yu, Haiyong Gu, Han B, Zhao H (2016). Surgical therapy for bilateral multiple primary lung cancer. Ann Thorac Surg.

[9] Hattori A, Takamochi K, Shiaki Oh, Suzuki K (2020). Prognostic classification of multiple primary lung cancers based on a ground-glass opacity component. Ann Thorac Surg.

[10] Zhang C, Yin K, Liu SY, Yan LX, Su J, Wu YL, Zhang XC, Zhong WZ, Yang XN. Multiomics analysis reveals a distinct response mechanism in multiple primary lung adenocarcinoma after neoadjuvant immunotherapy. J Immunother Cancer. 2021;9(4). 10.1136/jitc-2020-002312 PMC8025811 33820821

[11] Roth A, Khattra J, Yap D, Wan A, Laks E, Biele J, Ha G, Aparicio S, Bouchard-Côté A, Shah SP (2014). Pyclone: statistical inference of clonal population structure in cancer. Nat Methods.

[12] Pfeifer GP, Denissenko MF, Olivier M, Tretyakova N, Hecht SS, Hainaut P (2002). Tobacco smoke carcinogens, DNA damage and P53 mutations in smoking-associated cancers. Oncogene.

[13] Hattori A, Matsunaga T, Takamochi K, Shiaki Oh, Suzuki K (2017). Surgical management of multifocal ground-glass opacities of the lung: correlation of clinicopathologic and radiologic findings. Thorac Cardiovasc Surg.

[14] Chen TF, Xie CY, Rao BY, Shan SC, Zhang X, Zeng B, Lei YY, Luo HH (2019). Surgical treatment to multiple primary lung cancer patients: a systematic review and meta-analysis. BMC Surg.

[15] Zhang Y, Deng C, Ma X, Gao Z, Wang S, Zheng Q, Xia G, Wen Z, Han H, Fu F, Liu Q, Hu H, Li Y, Wong KK, Chen H (2020). Ground-glass opacity-featured lung adenocarcinoma has no response to chemotherapy. J Cancer Res Clin Oncol.

[16] Cai C, Cooper GF, Lu KN, Ma X, Xu S, Zhao Z, Chen X, Xue Y, Lee AV, Clark N, Chen V. Systematic discovery of the functional impact of somatic genome alterations in individual tumors through tumor-specific causal inference. PLoS computational biology. 2019;15(7):e1007088. 10.1371/journal.pcbi.1007088 PMC6650088 31276486

[17] Fang Y, Wang Y, Zeng D, Zhi S, Shu T, Huang Na, Zheng S, Jianhua Wu, Liu Y, Huang G, Xue Y, Bin J, Liao Y, Shi M, Liao W (2021). Comprehensive analyses reveal Tki-induced remodeling of the tumor immune microenvironment in Egfr/Alk-positive non-small-cell lung cancer. Oncoimmunology.

